# Chemical Analyses of Wasp-Associated *Streptomyces* Bacteria Reveal a Prolific Potential for Natural Products Discovery

**DOI:** 10.1371/journal.pone.0016763

**Published:** 2011-02-22

**Authors:** Michael Poulsen, Dong-Chan Oh, Jon Clardy, Cameron R. Currie

**Affiliations:** 1 Department of Bacteriology, University of Wisconsin-Madison, Madison, Wisconsin, United States of America; 2 Department of Biology, Section for Ecology and Evolution, University of Copenhagen, Copenhagen, Denmark; 3 Department of Biological Chemistry and Molecular Pharmacology, Harvard Medical School, Boston, Massachusetts, United States of America; 4 Natural Products Research Institute, College of Pharmacy, Seoul National University, Seoul, Republic of Korea; University of California Davis, United States of America

## Abstract

Identifying new sources for small molecule discovery is necessary to help mitigate the continuous emergence of antibiotic-resistance in pathogenic microbes. Recent studies indicate that one potentially rich source of novel natural products is Actinobacterial symbionts associated with social and solitary Hymenoptera. Here we test this possibility by examining two species of solitary mud dauber wasps, *Sceliphron caementarium* and *Chalybion californicum*. We performed enrichment isolations from 33 wasps and obtained more than 200 isolates of *Streptomyces* Actinobacteria. Chemical analyses of 15 of these isolates identified 11 distinct and structurally diverse secondary metabolites, including a novel polyunsaturated and polyoxygenated macrocyclic lactam, which we name sceliphrolactam. By pairing the 15 *Streptomyces* strains against a collection of fungi and bacteria, we document their antifungal and antibacterial activity. The prevalence and anti-microbial properties of Actinobacteria associated with these two solitary wasp species suggest the potential role of these *Streptomyces* as antibiotic-producing symbionts, potentially helping defend their wasp hosts from pathogenic microbes. Finding phylogenetically diverse and chemically prolific Actinobacteria from solitary wasps suggests that insect-associated Actinobacteria can provide a valuable source of novel natural products of pharmaceutical interest.

## Introduction

Small molecules derived from natural sources play a key role in human welfare, serving as drugs or drug precursors with useful pharmaceutical properties [1]–[2]. Among these natural products, antibiotics are particularly important, abating human suffering and death from infectious disease [1], [3]. The ability of microbes to rapidly evolve resistance compromises the efficacy of antibiotics, necessitating the continuous discovery of structurally novel natural products with antimicrobial properties [e.g., [3]–[5]]. However, traditional sources for discovering novel antibiotics appear to be largely exhausted [3], [6]. For example, contemporary bioprospecting of soil Actinobacteria, the most significant source of new antibiotics in the twentieth century, largely results in the rediscovery of already known compounds [e.g., [3], [6]–[7]]. Current strategies for addressing the urgent need for new antibiotics include metabolic engineering, synthetic chemistry, and genomic or metagenomic approaches [2], [7]–[13]. Another approach is identifying sources of microbes that have not been explored for their potential natural products [5]–[6], [14]–[21].

Symbiotic microbes may represent a particularly promising source because microbial symbioses are widespread [e.g., [22]], largely unexplored for natural products [cf. [5]; but see [16]], and often involve the exchange of small molecules between symbionts and hosts [cf. [16]], including compounds mediating host defense [e.g., [11], [17]–[19]]. Examples of novel small molecules from symbiotic microbes include pederins produced by uncultured endosymbiont bacteria associated with beetles and sponges [8], [11], [23], stilbene-derivatives produced by *Photorhabdus luminescens* associating with *Heterorhabditis* nematodes [24]–[25], and a number of compounds with antimicrobial properties derived from bacteria associated with bryozoans [reviewed by [26]]. Among symbiotic associations, insect-microbe symbioses that involve Actinobacteria may be of particular interest in natural product discovery. Recently, a *Streptomyces*-derived novel compound was discovered, mycangimycin, that appears to be responsible for the selective inhibition of an antagonistic fungus of the mutualistic fungus associated with *Dendroctonus frontalis* beetles [17], [19]. Similarly, the novel compound dentigerumycin was obtained from a fungus-growing ant-associated actinobacterium (genus *Pseudonocardia*), which aids in the protection of the ants' mutualistic fungus from a specialized parasite [18], [27], [28].

**Figure 1:**
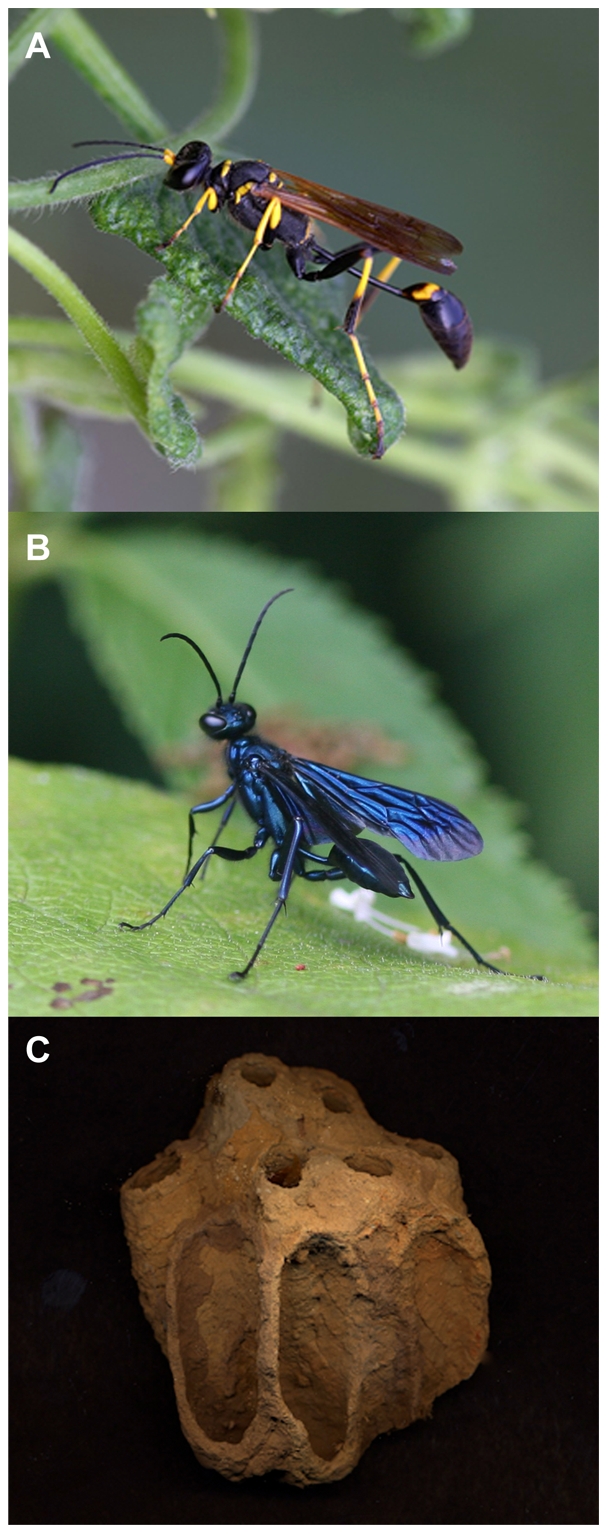
The two mud dauber species from which *Streptomyces* were obtained. A) shows a black and yellow mud dauber (*Sceliphron caementarium*) (courtesy of Linda Hendry), B) shows a blue mud dauber (*Chalybion californicum*) (courtesy of Patrick Belardo), and C) shows a nest of *Sceliphron caementarium* (courtesy of Jay King).

Here we examine if solitary wasp-associated Actinobacteria can serve as a valuable source of novel natural products. Specifically, we explore whether two species of solitary mud dauber wasps harbor Actinobacteria, and, if so, whether these bacteria produce bioactive natural products of ecological and pharmaceutical interests. We focused on these wasps, in part because of the recently discovered symbiosis between *Streptomyces* and 27 species of congeneric solitary European beewolf (genus *Philanthus*) wasps, where the bacteria are believed to produce antibiotics inhibiting fungal pathogens of the wasp larvae [29]–[31]. ‘Mud daubers’, as they are commonly referred to, belong to the families Sphecidae and Crabronidae, with the most common species in the US being the black and yellow mud dauber (*Sceliphron caementarium*, family Sphecidae) and the blue mud dauber (*Chalybion californicum*, family Sphecidae) (Fig. 1) [e.g., [32]]. *Sceliphron caementarium* mud daubers construct nests that are typically tubular and partitioned into multiple cells using mud collected from water puddles (Fig. 1) [e.g., [33]]. Individual cells contain a mud dauber egg and paralyzed prey (typically spiders), which serves as food for the developing larvae [32]. In contrast, *C. californicum* typically takes over an already established mud dauber nest, empties the contents of individual cells, and places its own paralyzed prey and eggs. Regardless of their life cycle, both mud dauber species frequently interact with the soil and prey insects, exposing both adults and brood to potentially pathogenic microbes.

Here we demonstrate that enrichment isolations for *Streptomyces* spp. from two species of mud dauber wasps can provide morphologically, phylogenetically, and chemically diverse Actinobacteria. Chemical characterization of the secondary metabolites produced revealed distinct and structurally diverse secondary metabolites, including a novel polyunsaturated and polyoxygenated macrocyclic lactam. Further, we assess the antibiotic properties of *Streptomyces* strains in antifungal and antibacterial Petri plate bioassays. We discuss our findings in relation to the potential role of *Streptomyces* in associations with solitary wasps, and suggest that our findings support that insect-associated Actinobacteria are a valuable source for novel natural products.

## Materials and Methods

### Collections and microbial isolations

Thirty-three individuals of two solitary wasp species, 25 *Sceliphron caementarium* (black and yellow mud daubers) and 8 *Chalybion californicum* (blue mud daubers), were collected in Madison, Wisconsin, in July 2006 and July 2007. Wasps were collected using sterile forceps while they were in the process of collecting mud by a small pond. Initially, each individual was washed in 500 µl sterile water to obtain Actinobacteria from the cuticle. Subsequently, individual wasps were divided into head, thorax, and abdomen, and each body part was ground separately in 500 µl sterile water. 200 µl of either the wash or the ground insect body part suspension was plated on each of two Petri plates with chitin medium containing antifungals (nystatin 10,000 units/mL and cycloheximide 5% w/v). Plates were stored for three weeks at 25°C, after which *Streptomyces* colony forming units were sub-cultured onto yeast malt extract agar (YMEA; 4 g/L yeast extract, 4 g/L dextrose, 10 g/L malt extract and 20 g/L agar) with antifungals (concentrations as above) [17]. One strain from each of 15 of the 24 obtained *Streptomyces* morphotypes, determined by growth pattern on YMEA medium (images shown in Fig. S1), was chosen for further analyses.

### Phylogenetic placement of *Streptomyces* strains

**Figure 2:**
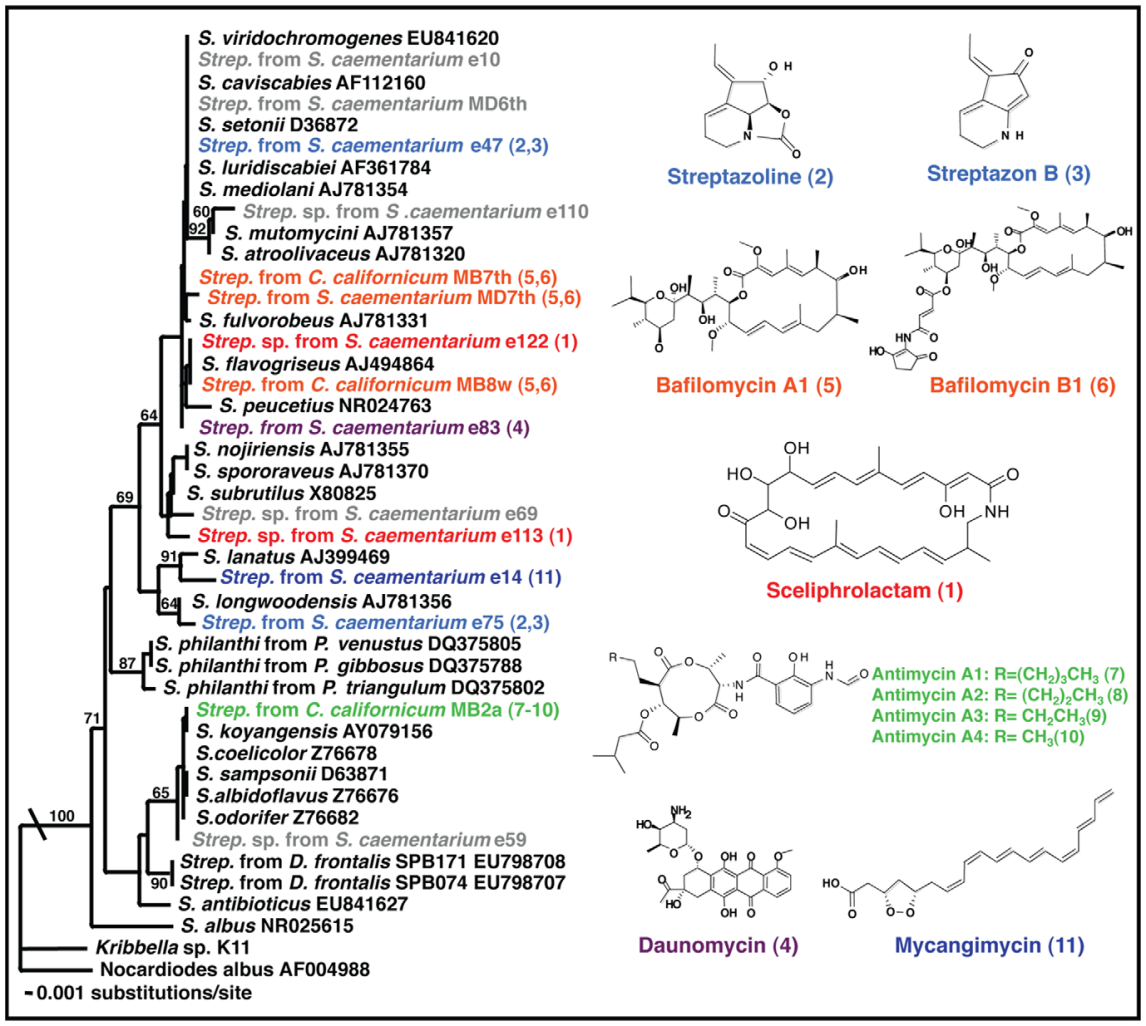
Phylogenetic and chemical diversity of mud dauber-associated *Streptomyces*. The phylogenetic placement based on partial *16S rDNA* sequences of the 15 *Streptomyces* strains examined for their secondary metabolites. The tree is rooted with *Kribbela* (GenBank accession number: GQ351313) and *Nocardioides* (GenBank accession numbers: AF004988). Mycangimycin-producing *Streptomyces* strains isolated from *Dendroctonus frontalis* (SPB071 and SPB074) and three *Streptomyces* strains (*S. philanthi*) from European Beewolves (*Philanthus venustus*, *P. gibbosus*, and *P. triangulum*) are included [17], [29]. Branch values indicate bootstrap support (>50 are given) of 1000 pseudoreplicates under Maximum Parsimony. The right panel shows the structures and names of the secondary metabolites identified, including the structurally novel macrocyclic lactam, scheliphrolactam. Strain names indicate the wasp species isolation source, which, as well as the compound names, are color-coded according to the compounds produced. Compounds could not be identified from strains indicated in grey font.

DNA extraction, PCR amplification and DNA sequencing of bacterial isolates were carried out according to procedures in Poulsen et al. [34]. Near full length sequences of *16S rRNA* were obtained from PCR using universal primers [27F and 1492R; [35]]. Positive bands on a 1.5% agarose gel were direct-sequenced at the University of Wisconsin-Madison Biotechnology Center (www.biotech.wisc.edu). Sequences were corrected for mismatches using Sequencher 4.6 for Windows (Gene Codes Corporation, Ann Arbor, MI), and the sequences were submitted to GenBank (Accession numbers: GQ351298-GQ351312) (Fig. S1). The wasp-associated strains were placed phylogenetically by performing a phylogenetic analysis that included the closest matches for each strain obtained in a type-strain search in the Ribosomal Database Project (http://rdp.cme.msu.edu/; [36] (Figs. 2; S1). The phylogeny was generated using PAUP* [37], after automatic and manual alignment using Clustal X [38] and MacClade [39], respectively.

### Chemical analysis of *Streptomyces* strains

The *Streptomyces* strains were cultivated in 25 mL of YMEB medium for 2–3 days and inoculated to 200 mL of YPM (2 g yeast extract, 2 g peptone, and 4 g mannitol per 1 L). The cultures were shaken at 250 rpm at 30°C. On days 2–7, 10 mL of cultures were extracted with ethyl acetate and organic extracts were prepared by evaporating ethyl acetate *in vacuo*. Dry material was resuspended in 0.5 mL of methanol and 10 µL of methanol solutions were injected into LC/MS for initial analysis (Agilent 1200 series HPLC/6130 mass spectrometer, Phenomenex C_18_(2) 4.6 mm×100 mm, 10–100% aqueous CH_3_CN with 0.1% formic acid over 20 min). Detected peaks in LC/MS profiles were further analyzed by comparing the UV database interlinked with the LC/MS system and microbial secondary metabolite database, Antibase 2005. Daunomycin, bafilomycin A1 and B1, and antimycin A1-A4 were purchased from Sigma Aldrich. ^1^H, ^13^C, and 2D NMR data were acquired on a Varian Inova 600 MHz spectrometer.

### Antifungal and antibacterial activities of *Streptomyces*-derived secondary metabolites

**Figure 3:**
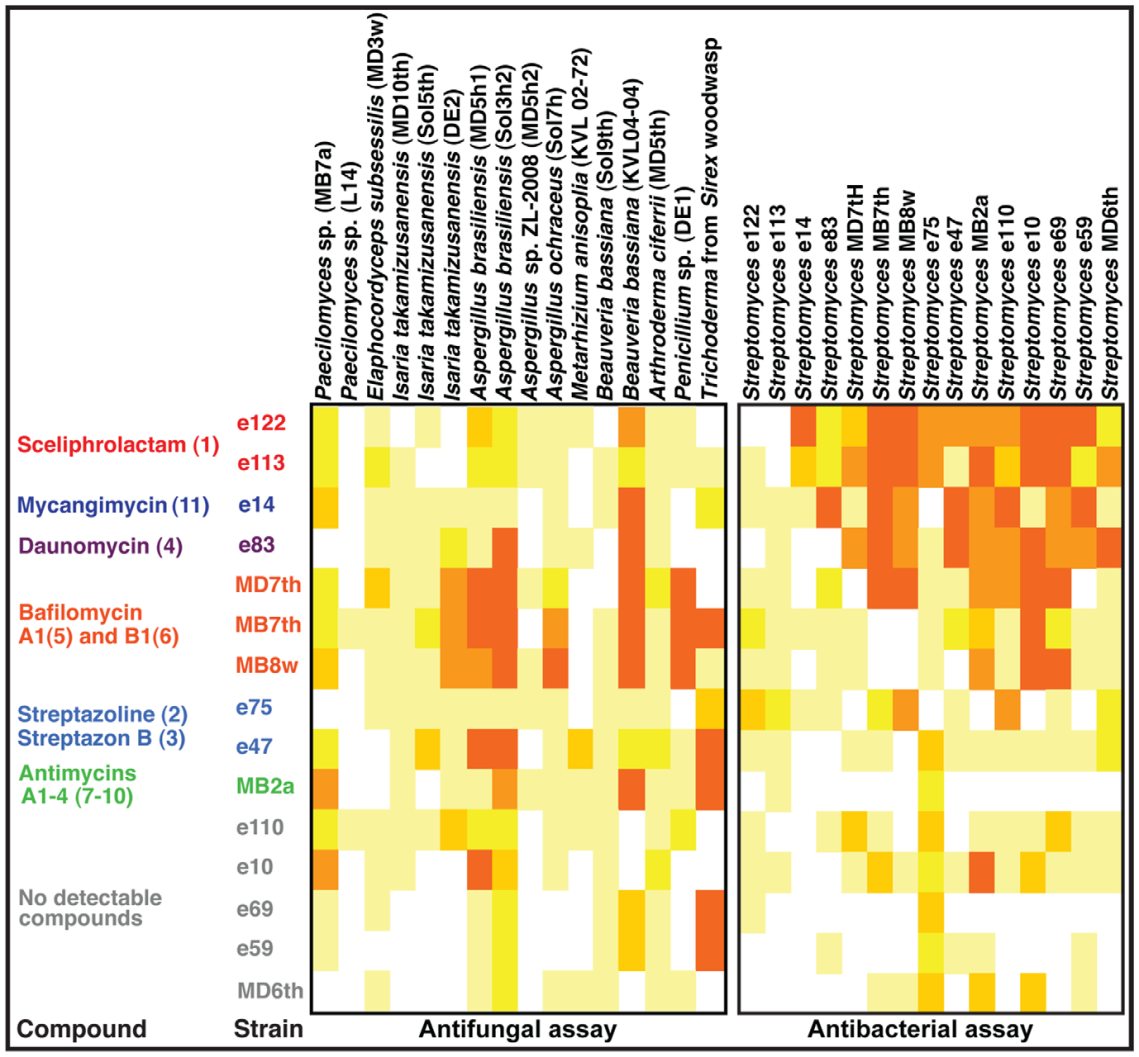
Bioassay evaluation of the antibiotic activity of the *Streptomyces* isolates from mud dauber wasps. Antimicrobial properties of *Streptomyces* in Petri plate boioassay tests against diverse fungi (left) and *Streptomyces* bacteria (right). Boxes represent average ZOI (n = 3) of a given pairing and different colors indicate the degree of inhibition. For *Streptomyces*-fungus bioassay: White: ZOI = 0 mm, Light yellow: ZOI = 0.01–1.0 mm, Yellow: ZOI = 1.01–2.0 mm, yellow-orange: ZOI = 2.01–3.0 mm, light orange: ZOI = 3.01–4.0, and orange: ZOI >4.01 mm. For *Streptomyces*-*Streptomyces* bioassay: White: ZOI = 0 mm, Light yellow: ZOI = 0.1–2.0 mm, Yellow: ZOI = 2.1–4.0 mm, yellow-orange: ZOI = 4.1–6.0 mm, light orange: ZOI = 6.1–8.0, and orange: ZOI >8.1 mm.

Antifungal and antibacterial activities were tested in two Petri plate bioassay experiments. The first experiment tested the antifungal effects of the 15 *Streptomyces* strains against 16 diverse fungi, including entomopathogens of insects, as well as a set of fungi isolated from mud daubers and *Sirex* wood wasps (Fig. 3). The identity of all fungi involved was confirmed by partial sequencing of 18S rDNA (not reported). Pure-culture *Streptomyces* were point inoculated on YMEA plates, left for three weeks until reaching a diameter of ca. 1.5 cm, after which the fungus was point inoculated at the edge of the plate. When a clear zone of inhibition (ZOI) had formed, typically within 2–3 weeks after fungal inoculation, the minimum distance was measured [cf. [28]]. Three replicates were performed for each pairing. The antibacterial assay was performed by pairing the 15 *Streptomyces* strains against each other in all possible combinations, thereby examining antibacterial properties against bacteria known to be present with the wasps. The inhibiting strain was point inoculated in the center of a Petri plate containing YMEA, and after three weeks the second strain was inoculated to the entire unoccupied plate area by applying 200 µl of autoclaved water containing 800–1200 cells/µl. The plates were checked bi-weekly until the second strain had either grown to fill the Petri plate or a distinct zone of inhibition (ZOI) had established [cf. Fig. 1 in [34]], which was then measured. Three replicates were performed for each pairing.

## Results

### Morphological and phylogenetic diversity of *Streptomyces* obtained from mud daubers

Targeted isolations of Actinobacteria from 25 black and yellow (*Sceliphron caementarium*) and eight blue (*Chalybion californicum*) mud daubers (Fig. 1) yielded more than 200 isolates of *Streptomyces*. Isolates were obtained from the head, thorax, and abdomen of all wasps as well as from whole-body washes. Based on isolate growth on nutrient-rich media, these strains could be grouped into 24 distinct morphotypes, 14 of which were obtained from individuals belonging to both wasp species, while five were unique to each species. Isolations from *S. caementarium* washes tended to yield the most morphotypes (Average 3.1 per wasp), while ground body parts yielded fewer morphotypes (Average 2.0, 2.1, and 1.8 from heads, thoraxes, and abdomens, respectively). Fewer morphological types (Average 1.0, 0.78, and 1.1 from heads, thoraxes, and abdomens, respectively) were obtained from *C. californicum*. A phylogenetic analysis conducted on 15 of the morphotypes (isolates chosen for chemical analyses, see below; Fig. S1) confirmed that these 15 strains represent diverse *Streptomyces* bacteria, with strains being distributed across much of the phylogeny of the genus (Figs. 2; S1). Some of the *Streptomyces* isolated from mud daubers claded together, including strains obtained from allospecific wasps; however, several strains were more closely related to *Streptomyces* not known to be associated with mud daubers (Figs. 2; S1).

### Chemical analysis of 15 mud dauber-associated *Streptomyces* strains

Chemical analyses of crude extracts from the 15 representative *Streptomyces* strains, using LC/MS, UV, and mass spectra, revealed diverse compounds produced by Actinobacteria obtained from mud daubers. In total, 11 different compounds were found from 10 of the 15 strains.

In the crude extracts from strains e113 and e122, we discovered a single major compound, named sceliphrolactam (**1**) (Fig. S2). The structure of sceliphrolactam is a previously unreported polyunsaturated and polyoxygenated 26-membered macrocyclic lactam [40]. No analogous compounds among published polyene macrocyclic lactams have been reported to share a similar carbon backbone. The most similar compounds, which are quite different from sceliphrolactam, are salinilactam from the marine actinomycete *Salinispora tropica* [41] and macromonosporin A from the acidic peat swamp forest Actinobacterium *Micromonospora* sp. [42].

In the strains e47 and e75, two major compounds were detected and identified as streptazoline (**2**) and streptazon B (**3**) by the UV database interlinked with LC/MS (Figs. 2; S3). The structures of these compounds, previously reported in *Streptomyces viridochromogenes* [43] and from an unidentified *Streptomyces* sp. [44], respectively, were confirmed by comparing ^1^H, ^13^C NMR, and mass spectra with the reported data [44]–[45] (Figs. S4–7). Daunomycin [4], previously isolated from *Streptomyces peucetius* [46], was detected in strain e83 by its characteristic UV (UV maxima 234, 254, 290, 476, and 496 nm) and mass spectra (Fig. S8), which were confirmed by comparison with authentic daunomycin (Fig. S9). Based on UV and mass spectra in the LC/MS analysis, we identified bafilomycin A1 (**5**) and B1 (**6**) from the strains MB8W, MD7th, and MB7th, previously reported in *Streptomyces griseus* [47] (Figs. 2; S10–11). The major secondary metabolites of the strain MB2a were determined as antimycins [7]–[10] (Fig. S12), which were previously reported from a soil *Streptomyces* sp. [48] (Fig. 2). These compounds were identified with UV and mass spectral analysis in LC/MS, and characterized by comparison to commercially available antimycins (Fig. S13). This comparison confirmed the structures of antimycin A1, A2, A3, and A4 (**7**–**10**) (Fig. 3). Lastly, the major secondary metabolite produced by strain e14 was identified as mycangimycin (**11**) by comparing the UV spectrum, the retention time, and the mass spectrum (Fig. S14) with the LC/MS profile of the southern pine beetle-associated mycangimycin-producer *Streptomyces* sp. SPB74 [17], [19] (Figs. 2; S15). The *Streptomyces* strains e10, e59, e69, e110, and MD6th did not produce secondary metabolites at levels detectable using the same culture conditions as those employed for the other 10 strains.

### Antifungal and antibacterial activities of *Streptomyces*-derived secondary metabolites

A potential ecological role for the secondary metabolites secreted by the *Streptomyces* sp. isolated from mud daubers is hygiene, inhibiting fungi or other bacteria in the environment. We tested this hypothesis by performing two Petri plate bioassay experiments. The first bioassay examined the antifungal effects of the 15 *Streptomyces* strains against diverse fungi, including known insect entomopathogic fungi, potential fungal parasites isolated from solitary wasps, and a *Trichoderma* sp. isolated from a *Sirex* wood wasp (Fig. 3). While most bacterial strains produced compounds with antifungal properties, there was abundant diversity in the degree and extent of inhibition (Fig. 3). Strains inhibited on average more than 10 fungi, although individual strains varied substantially in the number of fungi they suppressed (range 7–14). Several of the Actinobacteria, including strains producing different compounds, occasionally inhibited known insect entomopathogens (*Beauvaria* and *Metarhizium*), as well as potential insect pathogens (*Paecilomyces*, *Aspergillus*, and others) (Fig. 3). Strains producing the same compounds displayed similar, although never identical, inhibition profiles across the fungal strains (Fig. 3).

The second bioassay evaluated the diversity and extent of antibacterial properties of the *Streptomyces* spp. In order to perform this assay with ecologically relevant bacteria, i.e., ones known to be associated with the wasps, the assay evaluated inhibition of the 15 *Streptomyces* strains chosen in this study for phylogenetic and chemical characterization. This bioassay documented abundant variation in inhibitory capabilities, with the number of *Streptomyces* inhibited by individual strains being more variable than what was observed in the antifungal assay (average 10, range 2–14). As in the antifungal assay, strains with similar secondary metabolites displayed similar, but never identical, inhibition profiles (Fig. 3).

In our bioassays, the five *Streptomyces* strains from which no secondary metabolites were found, we did find some evidence for antimicrobial properties; these strains on average suppressed the growth of 9 (range 8–13) of the fungi and 7 (range 2–13) of the bacteria tested. However, overall this represented less general antimicrobial suppression than observed for the strains known to secrete secondary metabolites (growth suppression of an average of 12 (range 10–14) and 12 (range 2–14) fungi and bacteria, respectively) (Fig. 3). Further, the overall strength of inhibition in both assays tended to be lower in strains for which we did not detect compounds (Fig. 3).

## Discussion

Our findings show that diverse Actinobacteria in the genus *Streptomyces* can be readily isolated from a few individuals of a single insect group. Isolations from eight *C. californicum* and 25 *S. caementarium* yielded more than 200 strains of *Streptomyces* representing 24 distinct morphotypes. Fourteen of these morphotypes were isolated from individuals belonging to both wasp species, and five were unique to each wasp species. On average, individual *S. caementarium* wasps yielded more morphotypes than *C. californicum*; however, this is likely because three sub-cultures were performed per *C. californicum* isolation plate versus eight per *S. caementarium*. Our phylogenetic analysis of the 15 representative strains chosen for chemical analyses indicated that these strains represent a diverse collection of bacteria distributed across the genus *Streptomyces* (Figs. 2; S1), despite *16S rDNA* providing only limited phylogenetic resolution [e.g., [49]].

Our chemical analyses of the 15 *Streptomyces* strains revealed the production of a diverse collection of compounds. Ten of the 15 strains produce diffusible secondary metabolites from six structural classes: antimycins, bafilomycin A1 and B1, daunomycin, mycangimycin, streptazolin and streptazon B, and the previously unknown macrocyclic lactam, sceliphrolactam. As expected, different *Streptomyces* strains belonging to the same ’species’ can produce different secondary metabolites, while taxonomically diverse *Streptomyces* strains can produce identical metabolites. Embedded within the finding of diverse chemical compounds from the isolated *Streptomyces* bacteria was the discovery of sceliphrolactam: a structurally novel macrocyclic lactam produced by two genetically distinct *Streptomyces* strains (e113 and e122) (Fig. 2). Sceliphrolactam bears polyene and polyol moieties and could act as an antifungal by destabilizing the fungal cell membrane functions [cf., [50]]. Interestingly, none of the *Streptomyces* secreted compounds from more than one class *in vitro*, suggesting that individual strains produce a limited set of compounds within a single compound group under these conditions (Fig. 2).

Our Petri plate bioassay experiments against fungi and bacteria confirmed that different secondary metabolites are secreted by the *Streptomyces* strains, with strains varying in their antibacterial and antifungal activity (Fig. 3). Some of these differences were expected based on the known properties of previously reported compounds. For example, antimycins are potent inducers of cellular apoptosis in hepatocytes and bind to the hydrophobic groove of Bcl-2/Bcl-x proteins on the surface of mitochondria [51], which likely explains the antifungal activity (Fig. 3). Bafilomycins have both antifungal and cytotoxic properties [48], and they are potent inhibitors of vacuolar H^+^-ATPase [52]. Daunomycin inhibits DNA topoisomerase II and it thereby induces a cytotoxic antibiotic effect [53]. Mycangimycin is a selective antifungal agent, only recently obtained from a southern pine beetle-associated *Streptomyces* (Fig. 2) [17], [19]. While streptazolin itself showed limited antimicrobial activities [54], some of its derivatives, such as 3,9-dihydrostreptazolin, show enhanced antimicrobial and cytotoxic activities [43], which is consistent with the results of our assays. The strains producing these compounds mainly displayed weak or no inhibition of other Actinobacteria in bioassay, while their antifungal activity appeared restricted to a few *Aspergillus* strains and one *Trichoderma* strain (Fig. 3), suggesting narrow antimicrobial activity. Our antifungal assay revealed that the *Streptomyces* strains producing sceliphrolactam have antifungal properties (e.g., inhibiting certain strains of *Beauvaria*); however, they generally displayed stronger levels of inhibition of other *Streptomyces* bacteria than fungi (Fig. 3). Differences between strains known to produce the same compounds were observed, likely due to differences in concentrations of the compounds produced and/or due to differences in the relative composition of secretions in strains producing multiple compounds. Contrary to our expectations, we did observe antimicrobial properties of *Streptomyces* strains for which we did not detect secondary metabolites, suggesting that compounds indeed are produced and secreted by these strains (Fig. 3). Possible reasons for not detecting these compounds include secretion in concentrations below our detection threshold, or that compounds are not produced in pure culture, but elicited by the presence of another microbe.

The role of *Streptomyces* in the associations with mud dauber wasps remains enigmatic; they may be symbionts associated with the wasps or transient microbes picked up by wasps during soil interactions. Nevertheless, the tight association of solitary wasps with the soil, harboring an abundance of parasitic microbes, implies a potential benefit for the mud daubers of engaging in associations with antibiotic-producing bacteria. If so, *Streptomyces*-derived compounds with antimicrobial properties could serve to protect the wasps, the wasp brood, and/or help preserve the prey. *Streptomyces* presence on all body parts of all wasps sampled is a first indication that such a symbiotic association may be present; however, documenting the costs and benefits to the partners involved in this potential mutualism will be required [cf. [55]]. The only well-established *Streptomyces*-solitary wasp association involves the maintenance of the bacteria in antennal structures of European beewolf species, which benefit from the association by deriving antibiotic compounds that protect the wasp brood [29]-[31]. The mud daubers included in our study do not appear to have analogous antennal *Streptomyces*, and all wasp body parts yielded isolates, suggesting that *Streptomyces* are present at different, or less specialized, locations on the wasps.

If mud daubers benefit from Actinobacteria-derived compounds with antibiotic properties, they could either associate loosely with phylogenetically and chemically diverse *Streptomyces,* or more specifically with potentially coevolving *Streptomyces*. In fungus-growing ants, southern pine beetle insect-Actinobacteria, and European beewolves, one or a few specific Actinobacteria appear to associate with the host [17]–[19], [30]. In the European beewolf-*Streptomyces* association, the insect achieves protection through individual bacteria species secreting a mixture of antibiotic compounds [31]. In contrast, the diversity of *Streptomyces* found in this study suggests that the insect potentially takes advantage of multiple *Streptomyces* strains secreting diverse antibiotic compounds. Irrespective of whether mud daubers engage in specific defensive mutualisms with *Streptomyces* or not, the omnipresence of *Streptomyces* on all body parts of all wasps examined, coupled with the antibiotic properties of the bacteria-derived compounds, suggest that the presence of these *Streptomyces* sp. nevertheless affects the microbial communities associated with the wasps. This may include that compounds produced aid in competition with other bacteria, which is known to be frequent and important in shaping microbial communities [e.g., [56]].

Our findings of actinobacterial associations and identification of diverse bacterial secondary metabolites, especially the discovery of the novel macrocyclic lactam, sceliphrolactam, from mud dauber wasps demonstrate that actinobacterial communities from an insect host can aid in the search for novel natural products with antibiotic properties. Our analyses included only a subset of the *Streptomyces* we obtained from this insect group, suggesting that more compounds of natural product interest await discovery. Our search yielded a novel compound from chemical characterization of only 15 *Streptomyces* isolates, whereas searches for novel antibiotics among hundreds of soil Actinobacteria typically result in mainly rediscoveries of already known compounds [cf., [4]–[5], [7]]. Moreover, recent findings suggest that defensive mutualisms between insects and Actinobacteria are far more common than previously assumed [17]–[19], [29]–[30], [57], suggesting that insect-Actinobacteria associations are a particularly promising group from which novel natural products can be elicited. Future research should continue to identify novel insect-Actinobacteria associations, in addition to symbioses with bacteria beyond Actinobacteria [cf. [8], [16], [21]], including establishing the roles of the microbes and their secondary metabolites in the symbiotic systems. Such identification will both provide novel insight into defensive mutualisms and host-symbiont interactions in general, but also lead to the discovery of novel associations and novel secondary metabolites of natural product interest.

## Supporting Information

Figure S1 Morphological, genetic, and chemical diversity of *Streptomyces* isolated from black and yellow (*Sceliphron caementarium*) and blue (*Chalybion californicum*) mud daubers. Strain ID, wasp host, closest match in rdp (http://rdp.cme.msu.edu/; Cole et al. 2007) type strain searches, similarity scores, chemical compounds identified, and GenBank accession numbers are given for all isolates.
(PDF)

Figure S2 (a) LC/MS chromatograms of strain e113 (top) and e122 (bottom). UV spectra of the peak (sceliphrolactam) at 12.9 min in e113 (b) and in e122 (c). The ESI positive mode mass spectra of the peak (sceliphrolactam) at 13.0 min in e113 (d) and in e122 (e).
(PDF)

Figure S3 (a) The LC/MS chromatograms of strain e47 (top) and e75 (bottom). The UV spectra of the peaks at 7.5 min (streptazoline) (b) and at 7.8 min (streptazon B1) (c) in e47. The UV spectra of the peaks at 7.5 min (streptazoline) (d) and at 7.9 min (streptazon B1) (e).
(PDF)

Figure S4 ^1^H NMR spectrum of streptazoline (**2**) in CDCl_3_.
(PDF)

Figure S5 ^13^C NMR spectrum of streptazoline (**2**) in CDCl_3_.
(PDF)

Figure S6 ^1^H NMR spectrum of streptazon B (**3**) in CD_3_OD.
(PDF)

Figure S7 ^13^C NMR spectrum of streptazon B (**3**) in CD_3_OD.
(PDF)

Figure S8 (a) The LC/MS chromatogram of strain e83. Top: 210 nm trace. Bottom: Ion 528 trace. (b) The UV spectrum of the peak (daunomycin) at 8.6 min. (c) The ESI positive mode mass spectrum of the peak of daunomycin peak.
(PDF)

Figure S9 (a) The LC/MS chromatogram of authentic daunomycin. Top: 210 nm trace. Bottom: Ion 528 trace. (b) The UV spectrum of daunomycin at 8.7 min. (c) The ESI positive mode mass spectrum of the peak of daunomycin peak.
(PDF)

Figure S10 The LC/MS chromatograms of strains MB8W (a), MB7th (b), and MD7th (c). The UV spectra of the peak (bafilomycin A1) at 20.7 min (d) and the peak (bafilomycin B1) at 23.3 min (e) in MB7th. (f) The ESI positive mode mass spectrum of the bafilomycin A1 peak at 20.7 min.
(PDF)

Figure S11 The LC/MS chromatograms of bafilomycin A1 (a) and bafilomycin B1 (b). The UV spectra of bafilomycin A1 (c) and bafilomycin B1 (d). (e) The ESI positive mode mass spectrum of the bafilomycin A1.
(PDF)

Figure S12 (a) LC/MS chromatogram of strain MB2a. The UV spectra of the peaks at 21.3 min (antimycin A1) (b), at 20.3 min (antimycin A2) (c), at 19.6 (antimycin A3) (d), and at 18.5 (antimycin A4) (e). ESI negative mode MS of peaks of antimycin A1 (f), A2 (g), A3 (h), A4 (i).
(PDF)

Figure S13 (a) LC/MS chromatogram of antimycin A1, A2, A3, and A4. The UV spectra antimycin A1 (b), antimycin A2 (c), antimycin A3 (d), and antimycin A4 (e). The ESI negative mode mass spectra of the peaks of antimycin A1 (f), A2 (g), A3 (h), and A4 (i).
(PDF)

Figure S14 (a) The LC/MS chromatogram of strain e14. (b) The UV spectrum of the peak (mycangimycin) at 18.1 min. (c) The ESI positive mode mass spectrum of the peak of mycangimycin. (d) The ESI negative mode mass spectrum of the peak of mycangimycin.
(PDF)

Figure S15 (a) The LC/MS chromatogram of strain SPB74 (mycangimycin producer). (b) The UV spectrum of the peak (mycangimycin) at 18.2 min. (c) The ESI positive mode mass spectrum of the peak of mycangimycin. (d) The ESI negative mode mass spectrum of the peak of mycangimycin.
(PDF)
