# Supplementary material for: Chemical Analyses of Wasp-Associated Streptomyces Bacteria Reveal a Prolific Potential for Natural Products Discovery
Source: PLoS One. 2011 Feb 22;6(2):e16763. doi: 10.1371/journal.pone.0016763 (PMC3043073; doi:10.1371/journal.pone.0016763)
Supplement: Figure S1 — Morphological, genetic, and chemical diversity of Streptomyces isolated from black and yellow (Sceliphron caementarium) and blue (Chalybion californicum) mud daubers. Strain ID, wasp host, closest match in rdp (http://rdp.cme.msu.edu/; Cole et al. 2007) type strain searches, similarity scores, chemical compounds identified, and GenBank accession numbers are given for all isolates. (PDF) [file pone.0016763.s001.pdf]

Fig. S1. Morphological, genetic, and chemical diversity of *Streptomyces* isolated from black and yellow (*Sceliphron caementarium*) and blue (*Chalybion californicum*) mud daubers. Strain ID, wasp host, closest match in rdp (http://rdp.cme.msu.edu/; Cole et al. 2007) type strain searches, similarity scores, chemical compounds identified, and GenBank accession numbers are given for all isolates.

ID	Wasp host	Morphotype	Closest rdp type strain match (GenBank Acc. #)	Similarity	Compounds detected	GenBank Acc. #
e10	*S. caementarium*	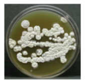	*S. setonii* (D63872), *S. caviscabies* (AF112160)	1.000	No compound detected	GQ351298
MD6th	*S. caementarium*	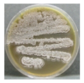	*S. caviscabies* (AF112160)	0.999	No compound detected	GQ351309
e47	*S. caementarium*	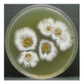	*S. setonii* (D63872), *S. caviscabies* (AF112160)	1.000	Streptazoline (2), Streptazone B (3)	GQ351300
e110	*S. caementarium*	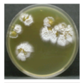	*S. caviscabies* (AF112160), *S. luridiscabeie* (AF361784) *S. atroolivaceus* (AJ781320), *S. mutomycini* (AJ781357)	0.989	No compound detected	GQ351305
MB7th	*C.californicum*	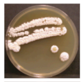	*S. fulvorobeus* (AJ781331)	0.997	Bafilomycin A1 (5), B1 (6)	GQ351311
e83	*S. caementarium*	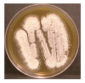	*S. caviscabies* (AF112160), *S. flavogriseus* (AJ494864)	0.999	Daunomycin (4)	GQ351304
MD7th	*S. caementarium*	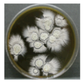	*S. mediolani* (AJ781354)	0.994	Bafilomycin A1 (5), B1 (6)	GQ351308
e122	S. caementarium	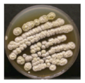	S. flavogriseus (AJ494864)	1.000	Sceliphrolactam (1)	GQ351307
MB8w	S. caementarium	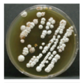	S. flavogriseus (AJ494864)	0.999	Bafilomycin A1 (5), B1 (6)	GQ351312
e69	S. caementarium	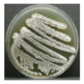	S. nojirensis (AJ781355), S. spororaveus (AJ781370)	0.993	No compound detected	GQ351302
e113	S. caementarium	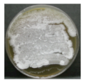	S. subrutilus (X80825)	0.996	Sceliphrolactam (1)	GQ351306
e14	S. caementarium	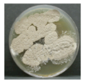	S. lanatus (AJ399469)	0.990	Mycangimycin (11)	GQ351299
e75	S. caementarium	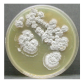	S. longwoodiensis (AJ781356)	0.996	Streptazoline (2), Streptazone B (3)	GQ351303
MB2a	C.californicum	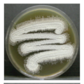	*S. koyangensis* (AY079159), *S. sampsonii* (D63871), *S. albidoflavus* (Z76676), *S. coelicolor* (Z76678), *S. odorifer* (Z76682)	0.999	Antimycin A1-4 (7-10)	GQ351310
e59	*S. caementarium*	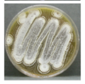	S. koyangensis (AY079159), S. sampsonii (D63871), S. albidoflavus (Z76676), S. coelicolor (Z76678), S. odorifer (Z76682)	0.999	No compound detected	GQ351301
