# Supplementary material for: Chemical Analyses of Wasp-Associated Streptomyces Bacteria Reveal a Prolific Potential for Natural Products Discovery
Source: PLoS One. 2011 Feb 22;6(2):e16763. doi: 10.1371/journal.pone.0016763 (PMC3043073; doi:10.1371/journal.pone.0016763)
Supplement: Figure S2 — (a) LC/MS chromatograms of strain e113 (top) and e122 (bottom). UV spectra of the peak (sceliphrolactam) at 12.9 min in e113 (b) and in e122 (c). The ESI positive mode mass spectra of the peak (sceliphrolactam) at 13.0 min in e113 (d) and in e122 (e). (PDF) [file pone.0016763.s002.pdf]

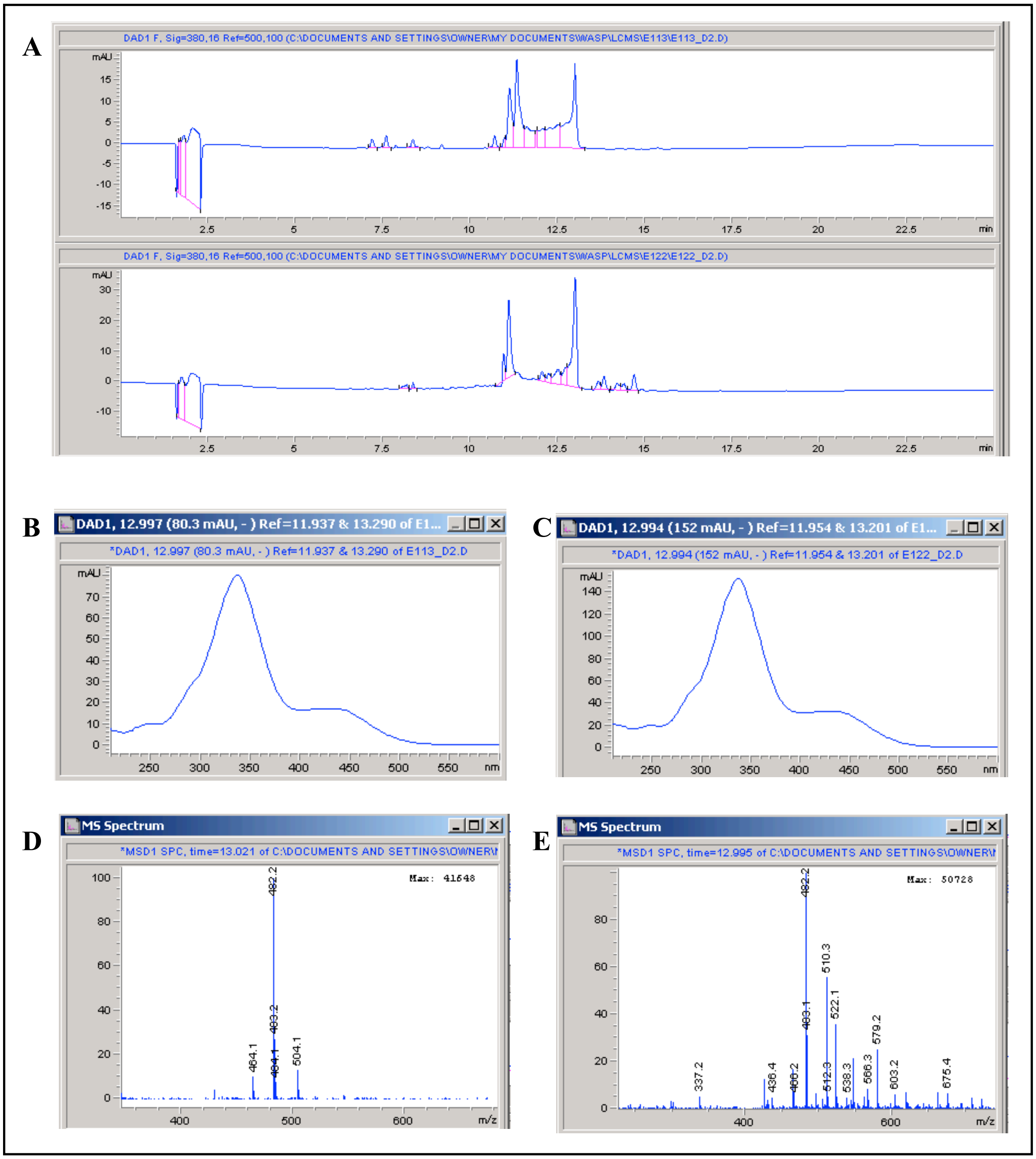

Fig. S2. (a) LC/MS chromatograms of strain e113 (top) and e122 (bottom). UV spectra of the peak (sceliphrolactam) at 12.9 min in e113 (b) and in e122 (c). The ESI positive mode mass spectra of the peak (sceliphrolactam) at 13.0 min in e113 (d) and in e122 (e).
