# Supplementary material for: Chemical Analyses of Wasp-Associated Streptomyces Bacteria Reveal a Prolific Potential for Natural Products Discovery
Source: PLoS One. 2011 Feb 22;6(2):e16763. doi: 10.1371/journal.pone.0016763 (PMC3043073; doi:10.1371/journal.pone.0016763)
Supplement: Figure S3 — (a) The LC/MS chromatograms of strain e47 (top) and e75 (bottom). The UV spectra of the peaks at 7.5 min (streptazoline) (b) and at 7.8 min (streptazon B1) (c) in e47. The UV spectra of the peaks at 7.5 min (streptazoline) (d) and at 7.9 min (streptazon B1) (e). (PDF) [file pone.0016763.s003.pdf]

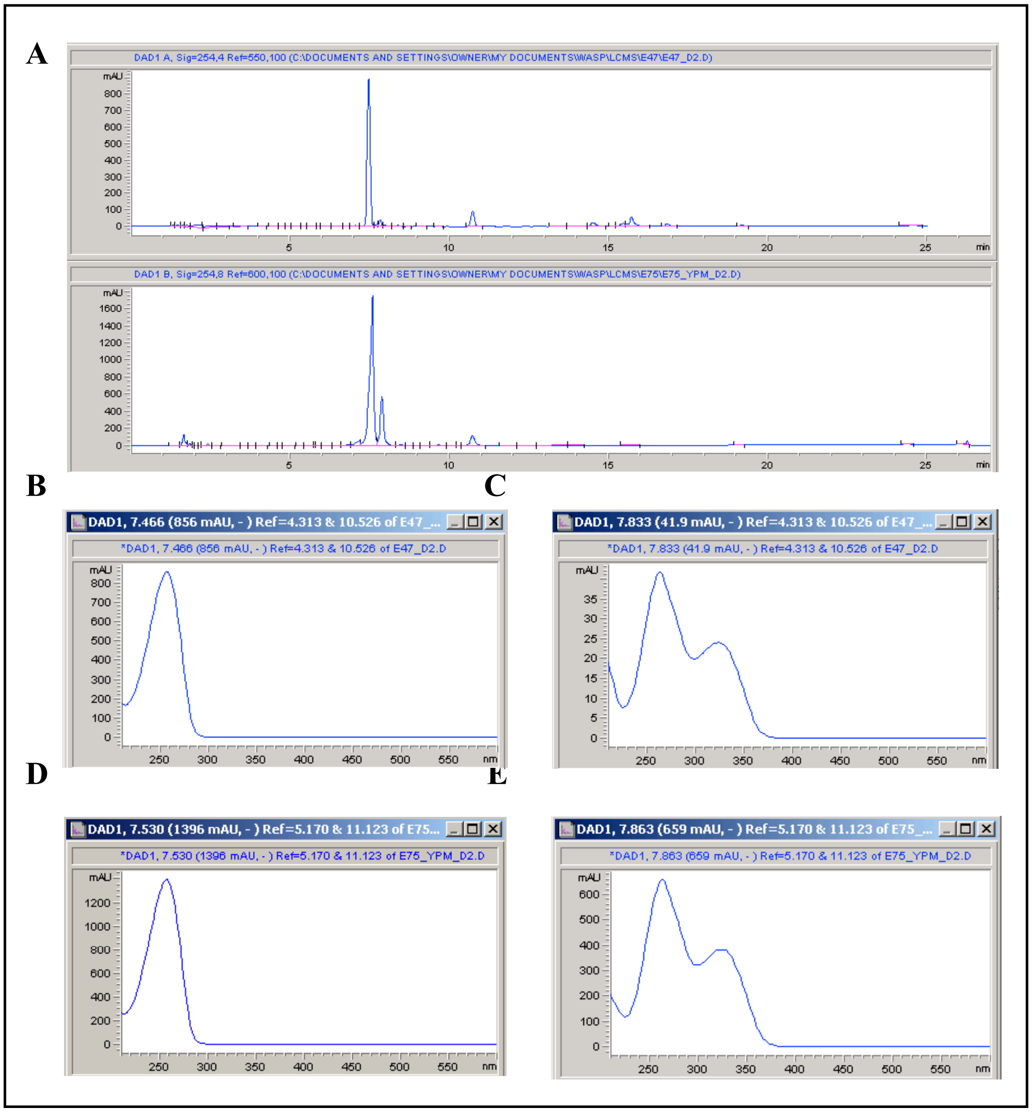

Fig. S3. (a) The LC/MS chromatograms of strain e47 (top) and e75 (bottom). The UV spectra of the peaks at 7.5 min (streptazoline) (b) and at 7.8 min (streptazon B1) (c) in e47. The UV spectra of the peaks at 7.5 min (streptazoline) (d) and at 7.9 min (streptazon B1) (e).
