# Supplementary figures and images for: Chemical Analyses of Wasp-Associated Streptomyces Bacteria Reveal a Prolific Potential for Natural Products Discovery
Source: PLoS One. 2011 Feb 22;6(2):e16763. doi: 10.1371/journal.pone.0016763 (PMC3043073; doi:10.1371/journal.pone.0016763)

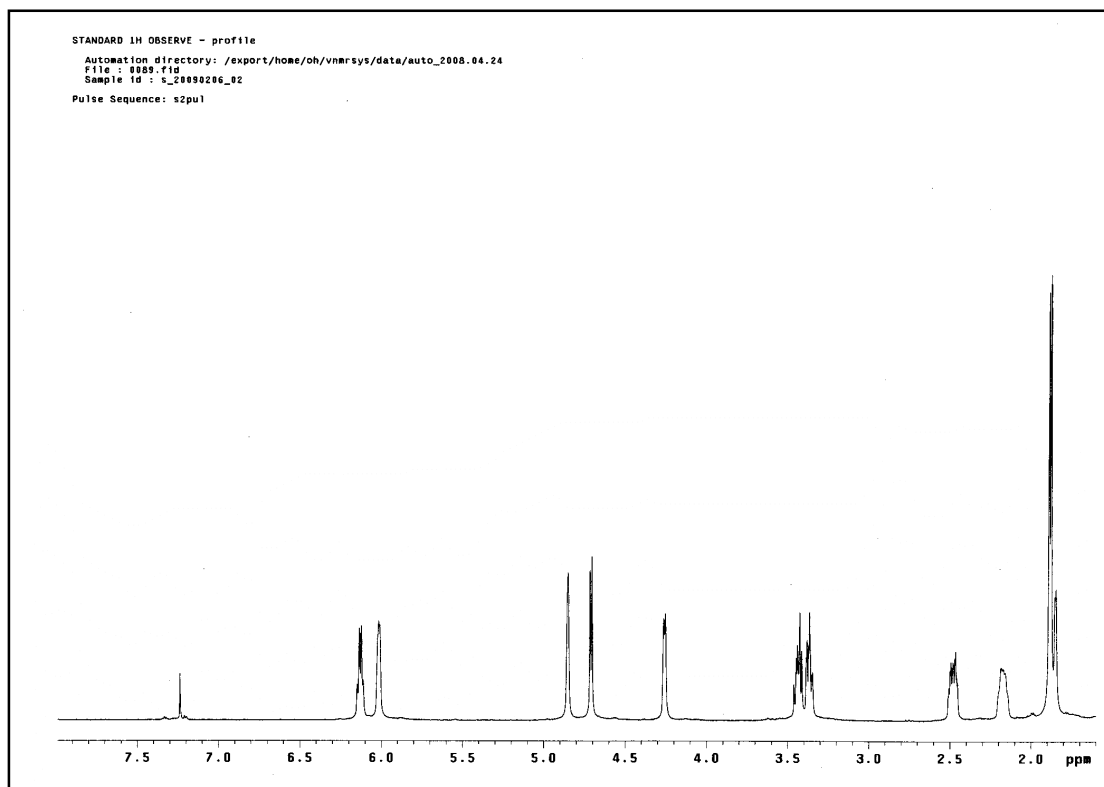

Fig.

Fig. S4. <sup>1</sup>H NMR spectrum of streptazoline (**2**) in CDCl<sub>3</sub>.

Supplement: Figure S4 — 1H NMR spectrum of streptazoline (2) in CDCl3. (PDF) [file pone.0016763.s004.pdf]

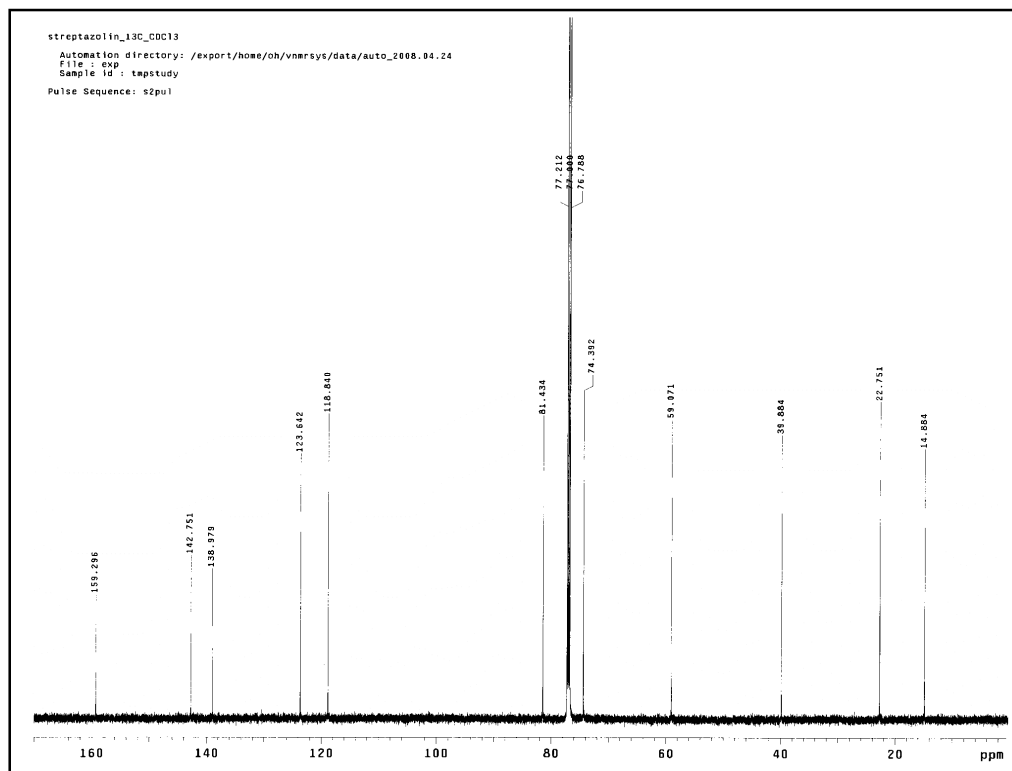

Fig. S5.  $^{13}\text{C}$  NMR spectrum of streptazolin (**2**) in  $\text{CDCl}_3$ .

Supplement: Figure S5 — 13C NMR spectrum of streptazoline (2) in CDCl3. (PDF) [file pone.0016763.s005.pdf]

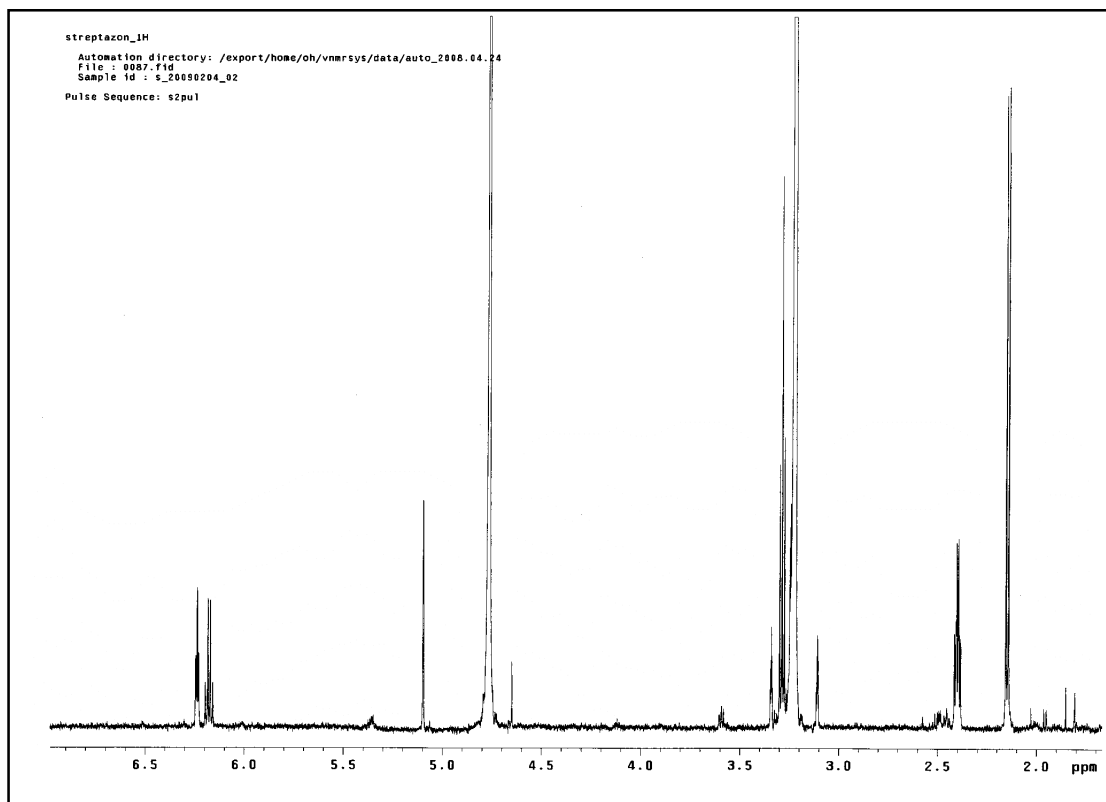

Fig. S6.  $^1\text{H}$  NMR spectrum of streptazon B (**3**) in  $\text{CD}_3\text{OD}$ .

Supplement: Figure S6 — 1H NMR spectrum of streptazon B (3) in CD3OD. (PDF) [file pone.0016763.s006.pdf]

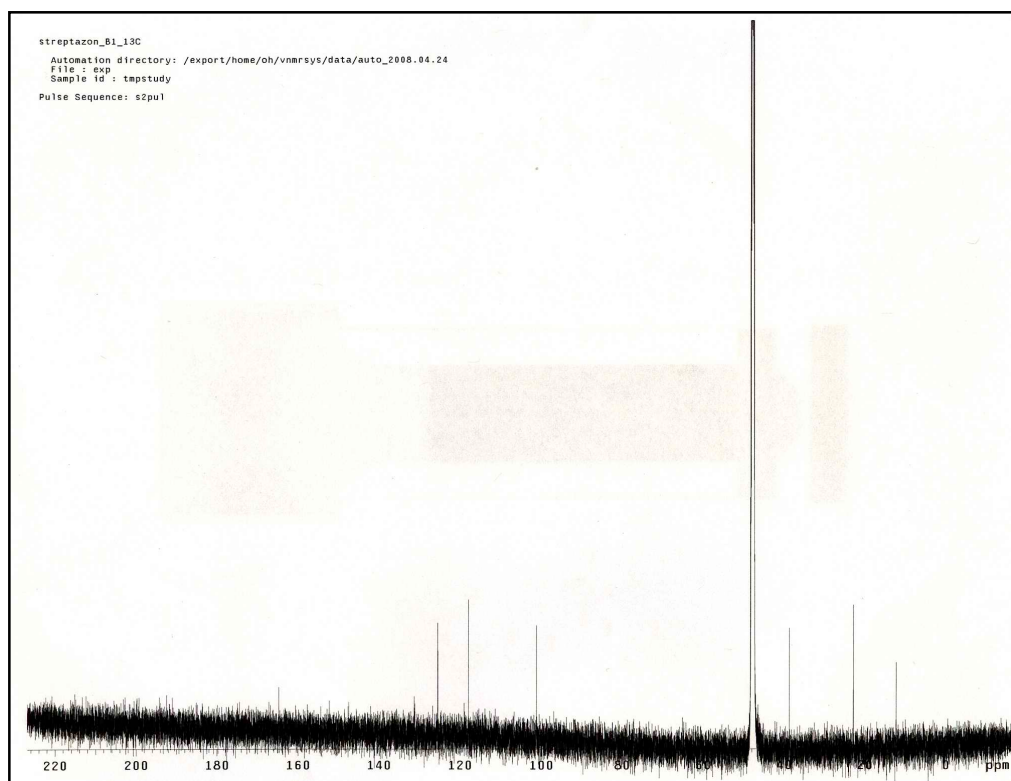

Fig. S7.  $^{13}\text{C}$  NMR spectrum of streptazon B (**3**) in  $\text{CD}_3\text{OD}$ .

Supplement: Figure S7 — 13C NMR spectrum of streptazon B (3) in CD3OD. (PDF) [file pone.0016763.s007.pdf]
