# Supplementary material for: Chemical Analyses of Wasp-Associated Streptomyces Bacteria Reveal a Prolific Potential for Natural Products Discovery
Source: PLoS One. 2011 Feb 22;6(2):e16763. doi: 10.1371/journal.pone.0016763 (PMC3043073; doi:10.1371/journal.pone.0016763)
Supplement: Figure S9 — (a) The LC/MS chromatogram of authentic daunomycin. Top: 210 nm trace. Bottom: Ion 528 trace. (b) The UV spectrum of daunomycin at 8.7 min. (c) The ESI positive mode mass spectrum of the peak of daunomycin peak. (PDF) [file pone.0016763.s009.pdf]

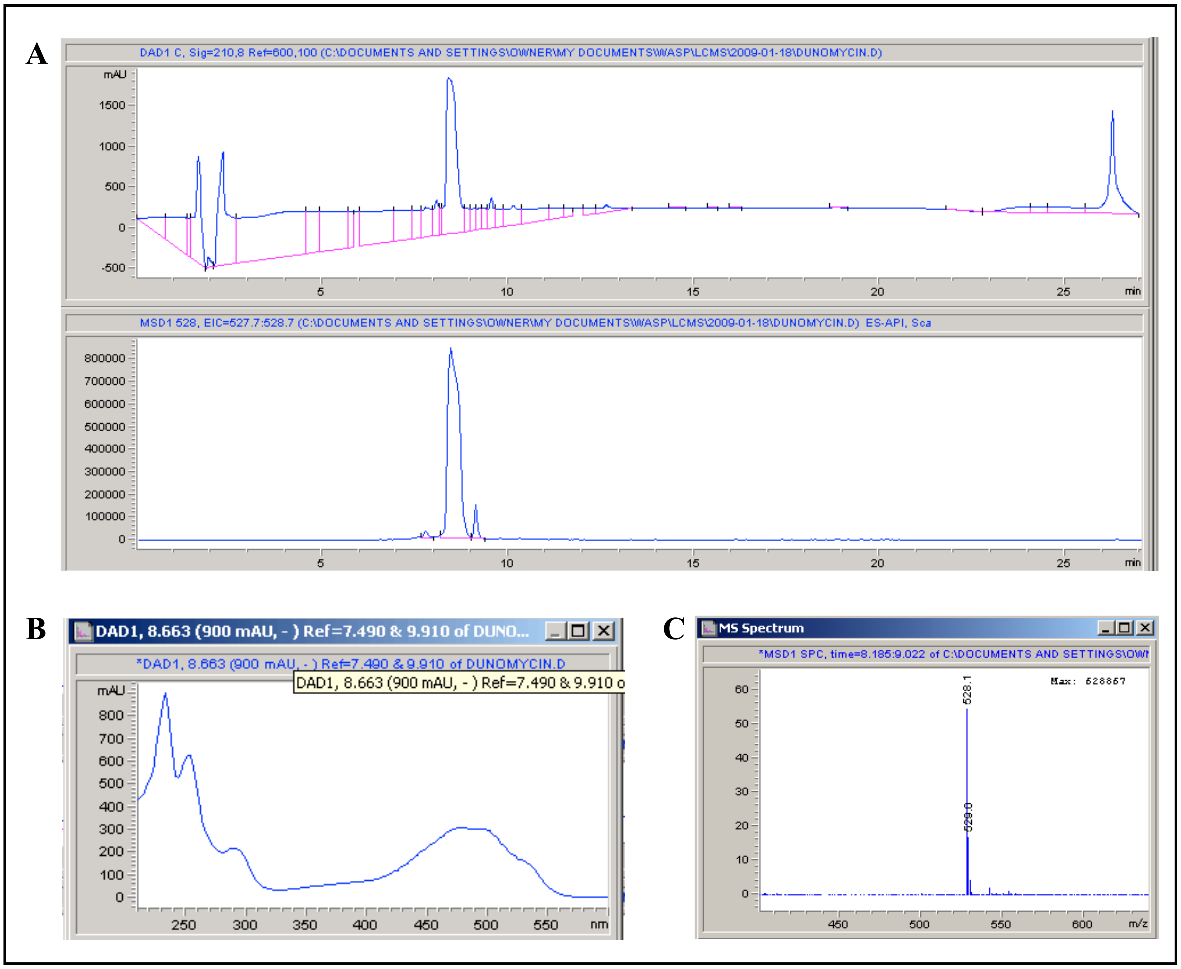

Fig. S9. (a) The LC/MS chromatogram of authentic daunomycin. Top: 210 nm trace. Bottom: Ion 528 trace. (b) The UV spectrum of daunomycin at 8.7 min. (c) The ESI positive mode mass spectrum of the peak of daunomycin peak.
