# Supplementary material for: Chemical Analyses of Wasp-Associated Streptomyces Bacteria Reveal a Prolific Potential for Natural Products Discovery
Source: PLoS One. 2011 Feb 22;6(2):e16763. doi: 10.1371/journal.pone.0016763 (PMC3043073; doi:10.1371/journal.pone.0016763)
Supplement: Figure S10 — The LC/MS chromatograms of strains MB8W (a), MB7th (b), and MD7th (c). The UV spectra of the peak (bafilomycin A1) at 20.7 min (d) and the peak (bafilomycin B1) at 23.3 min (e) in MB7th. (f) The ESI positive mode mass spectrum of the bafilomycin A1 peak at 20.7 min. (PDF) [file pone.0016763.s010.pdf]

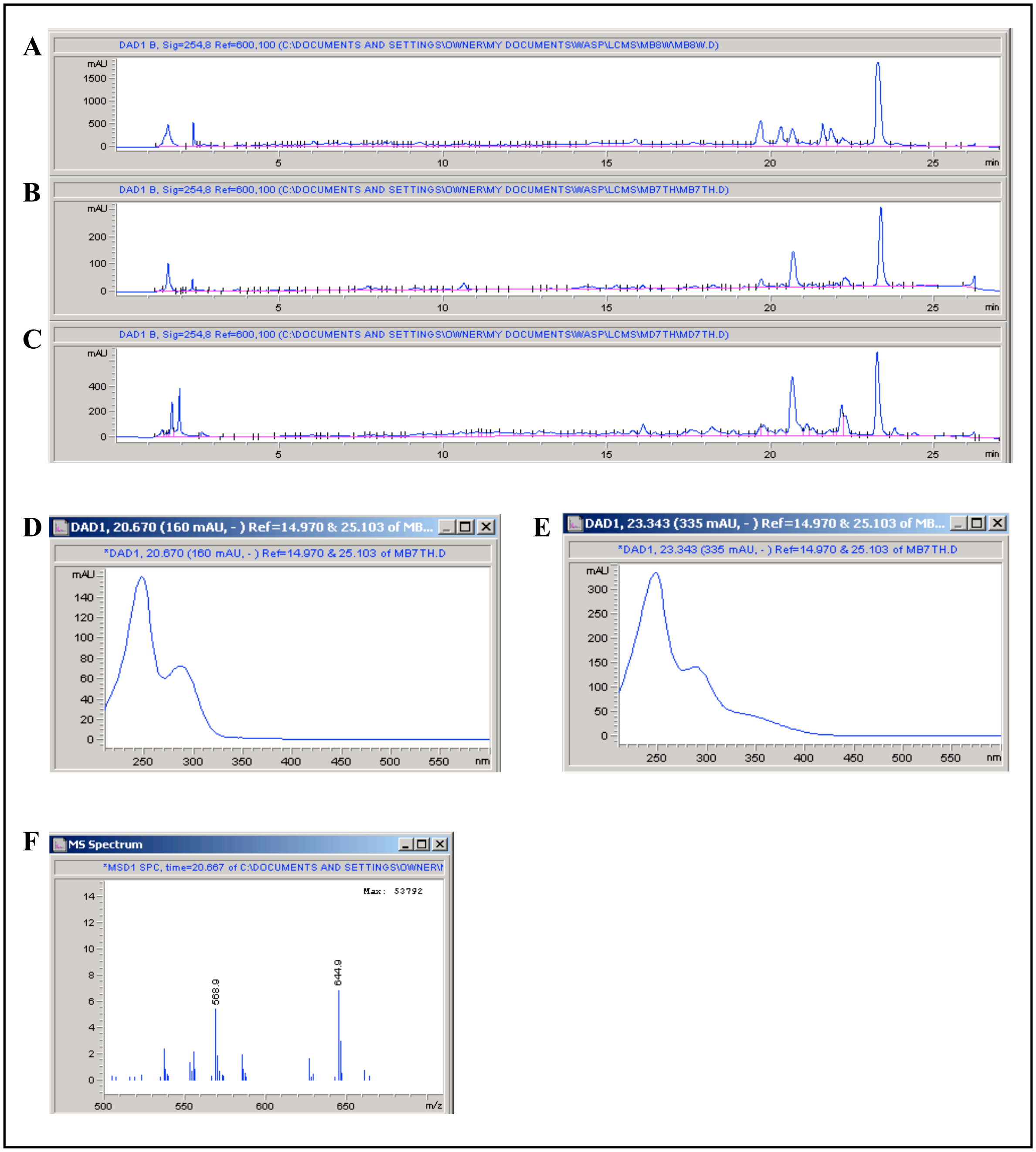

Fig. S10. The LC/MS chromatograms of strains MB8W (a), MB7th (b), and MD7th (c). The UV spectra of the peak (bafilomycin A1) at 20.7 min (d) and the peak (bafilomycin B1) at 23.3 min (e) in MB7th. (f) The ESI positive mode mass spectrum of the bafilomycin A1 peak at 20.7 min.
