# Supplementary material for: Chemical Analyses of Wasp-Associated Streptomyces Bacteria Reveal a Prolific Potential for Natural Products Discovery
Source: PLoS One. 2011 Feb 22;6(2):e16763. doi: 10.1371/journal.pone.0016763 (PMC3043073; doi:10.1371/journal.pone.0016763)
Supplement: Figure S11 — The LC/MS chromatograms of bafilomycin A1 (a) and bafilomycin B1 (b). The UV spectra of bafilomycin A1 (c) and bafilomycin B1 (d). (e) The ESI positive mode mass spectrum of the bafilomycin A1. (PDF) [file pone.0016763.s011.pdf]

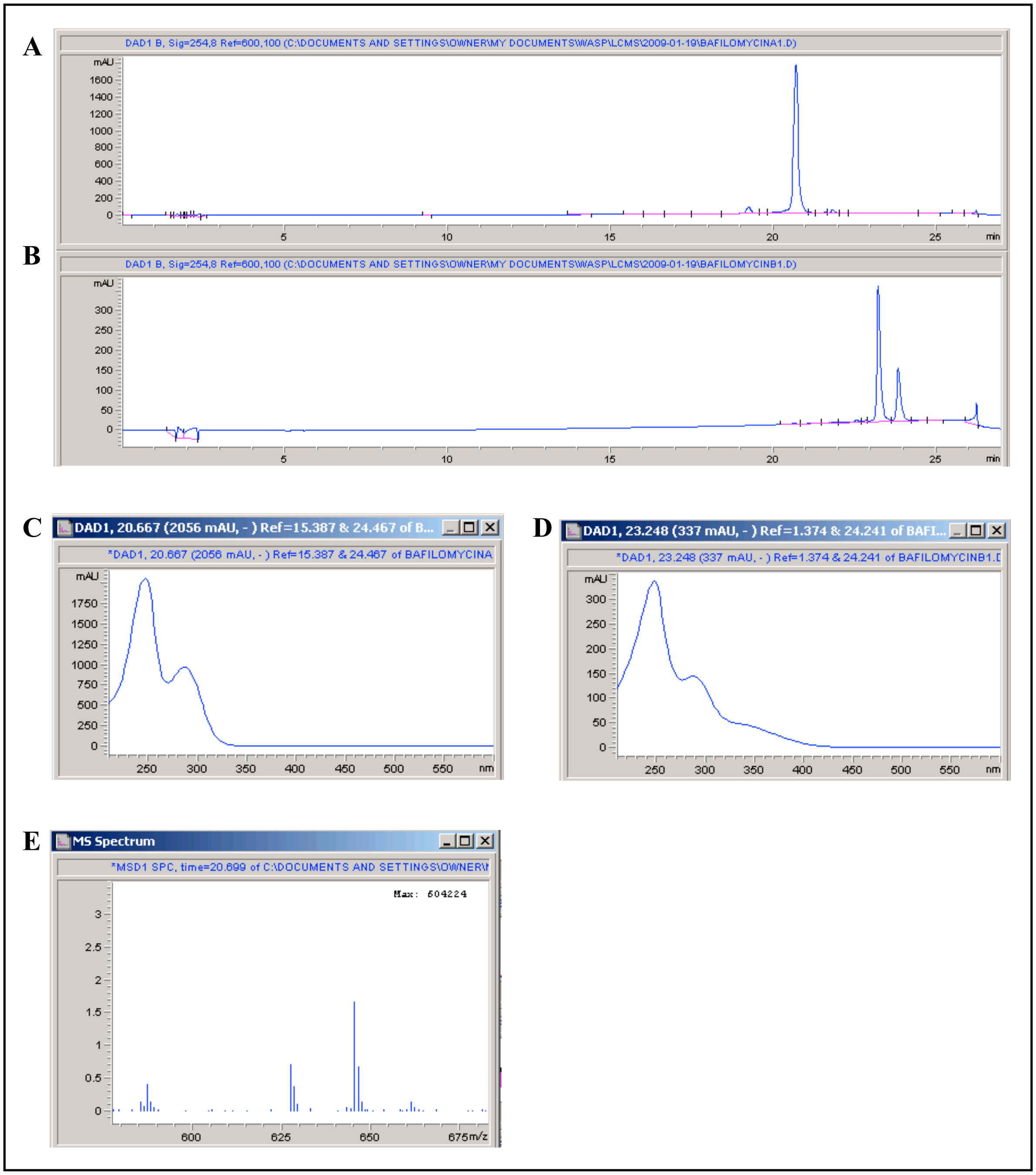

Fig. S11. The LC/MS chromatograms of bafilomycin A1 (a) and bafilomycin B1 (b). The UV spectra of bafilomycin A1 (c) and bafilomycin B1 (d). (e) The ESI positive mode mass spectrum of the bafilomycin A1.
