# Supplementary material for: Chemical Analyses of Wasp-Associated Streptomyces Bacteria Reveal a Prolific Potential for Natural Products Discovery
Source: PLoS One. 2011 Feb 22;6(2):e16763. doi: 10.1371/journal.pone.0016763 (PMC3043073; doi:10.1371/journal.pone.0016763)
Supplement: Figure S12 — (a) LC/MS chromatogram of strain MB2a. The UV spectra of the peaks at 21.3 min (antimycin A1) (b), at 20.3 min (antimycin A2) (c), at 19.6 (antimycin A3) (d), and at 18.5 (antimycin A4) (e). ESI negative mode MS of peaks of antimycin A1 (f), A2 (g), A3 (h), A4 (i). (PDF) [file pone.0016763.s012.pdf]

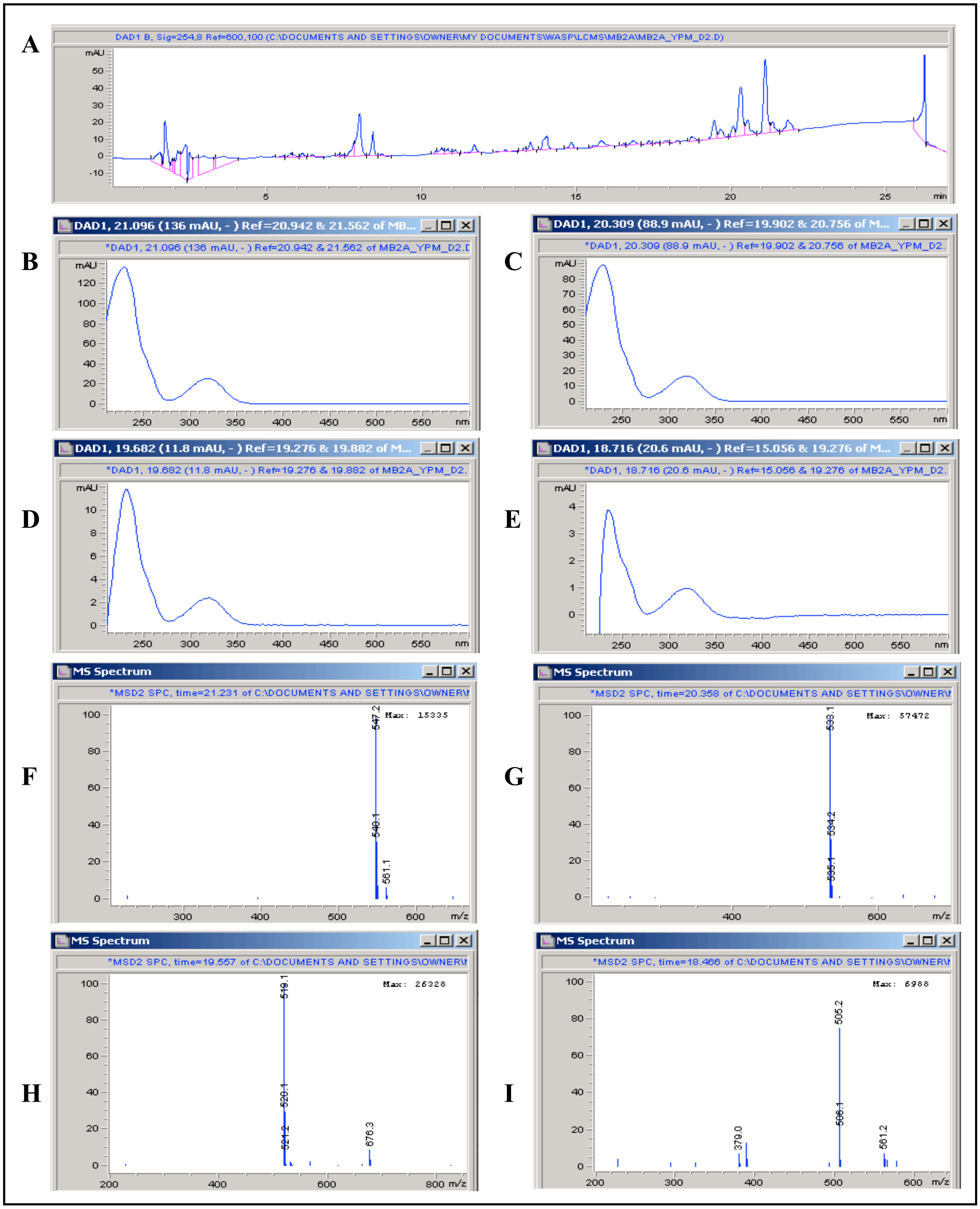

Fig. S12. (a) LC/MS chromatogram of strain MB2a. The UV spectra of the peaks at 21.3 min (antimycin A1) (b), at 20.3 min (antimycin A2) (c), at 19.6 (antimycin A3) (d), and at 18.5 (antimycin A4) (e). ESI negative mode MS of peaks of antimycin A1 (f) , A2 (g), A3 (h), A4 (i).
