# Supplementary material for: Chemical Analyses of Wasp-Associated Streptomyces Bacteria Reveal a Prolific Potential for Natural Products Discovery
Source: PLoS One. 2011 Feb 22;6(2):e16763. doi: 10.1371/journal.pone.0016763 (PMC3043073; doi:10.1371/journal.pone.0016763)
Supplement: Figure S13 — (a) LC/MS chromatogram of antimycin A1, A2, A3, and A4. The UV spectra antimycin A1 (b), antimycin A2 (c), antimycin A3 (d), and antimycin A4 (e). The ESI negative mode mass spectra of the peaks of antimycin A1 (f), A2 (g), A3 (h), and A4 (i). (PDF) [file pone.0016763.s013.pdf]

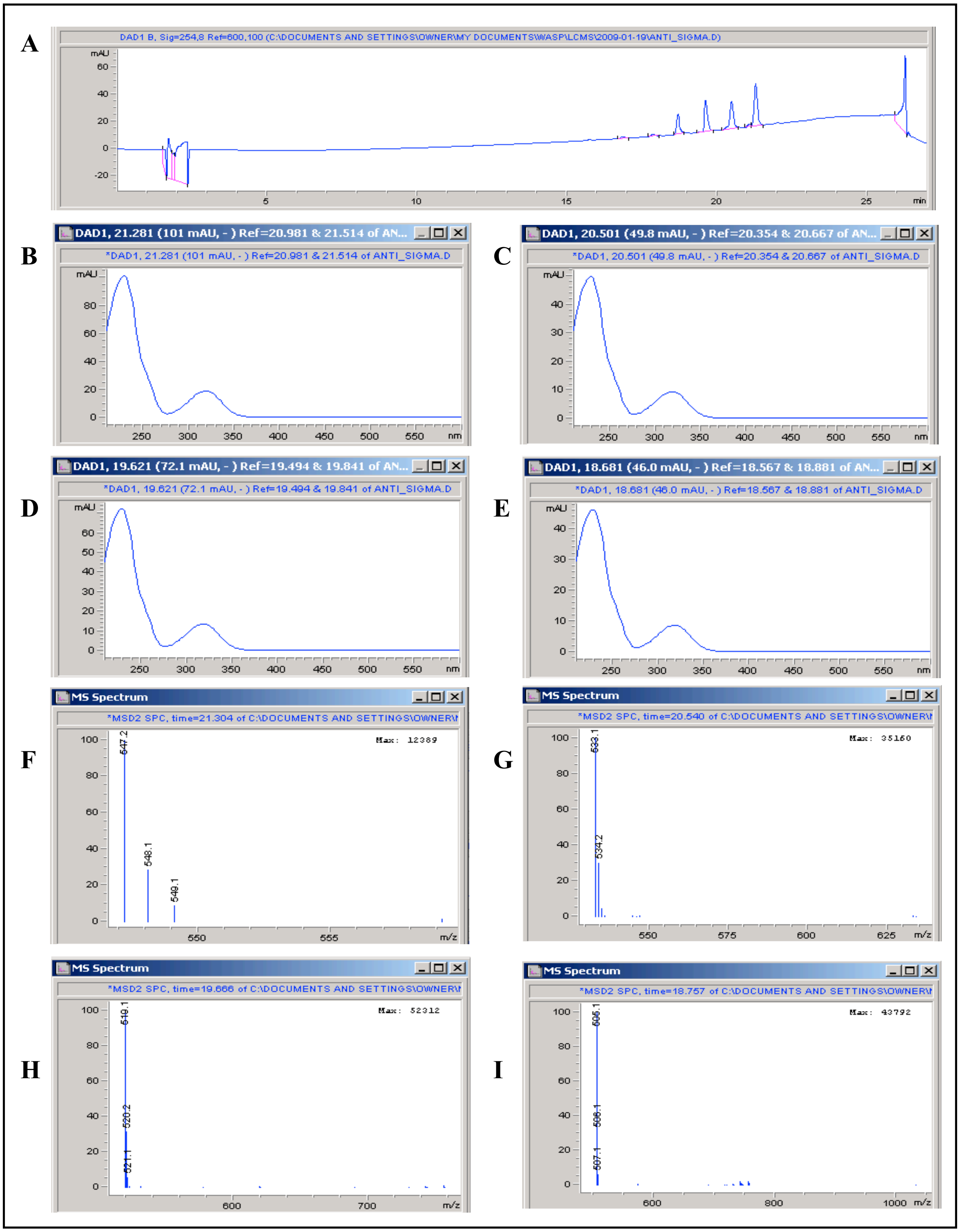


Fig. S13. (a) LC/MS chromatogram of antimycin A1, A2, A3, and A4. The UV spectra antimycin A1 (b), antimycin A2 (c), antimycin A3 (d), and antimycin A4 (e). The ESI negative mode mass spectra of the peaks of antimycin A1 (f) , A2 (g), A3 (h), and A4 (i).
