# Supplementary material for: Chemical Analyses of Wasp-Associated Streptomyces Bacteria Reveal a Prolific Potential for Natural Products Discovery
Source: PLoS One. 2011 Feb 22;6(2):e16763. doi: 10.1371/journal.pone.0016763 (PMC3043073; doi:10.1371/journal.pone.0016763)
Supplement: Figure S15 — (a) The LC/MS chromatogram of strain SPB74 (mycangimycin producer). (b) The UV spectrum of the peak (mycangimycin) at 18.2 min. (c) The ESI positive mode mass spectrum of the peak of mycangimycin. (d) The ESI negative mode mass spectrum of the peak of mycangimycin. (PDF) [file pone.0016763.s015.pdf]

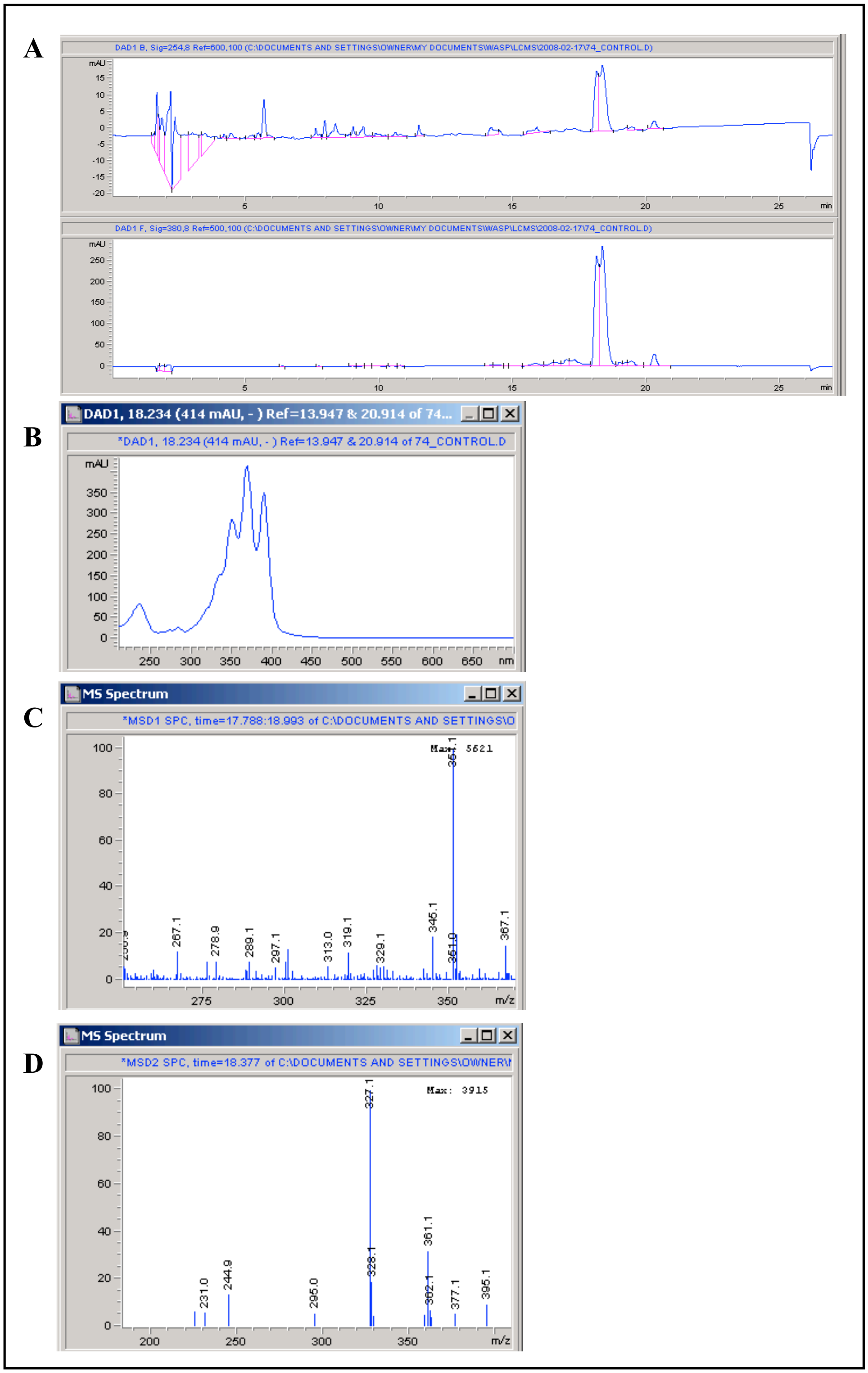

Fig. S15. (a) The LC/MS chromatogram of strain SPB74 (mycangimycin producer). (b) The UV spectrum of the peak (mycangimycin) at 18.2 min. (c) The ESI positive mode mass spectrum of the peak of mycangimycin. (d) The ESI negative mode mass spectrum of the peak of mycangimycin.
